# Virtual Electrophysiological Study of Atrial Fibrillation in Fibrotic Remodeling

**DOI:** 10.1371/journal.pone.0117110

**Published:** 2015-02-18

**Authors:** Kathleen S. McDowell, Sohail Zahid, Fijoy Vadakkumpadan, Joshua Blauer, Rob S. MacLeod, Natalia A. Trayanova

**Affiliations:** 1 The Johns Hopkins University, Department of Biomedical Engineering, Baltimore, Maryland, United States of America; 2 University of Utah, Comprehensive Arrhythmia Research and Management Center, School of Medicine, Salt Lake City, Utah, United States of America; Gent University, BELGIUM

## Abstract

Research has indicated that atrial fibrillation (AF) ablation failure is related to the presence of atrial fibrosis. However it remains unclear whether this information can be successfully used in predicting the optimal ablation targets for AF termination. We aimed to provide a proof-of-concept that patient-specific virtual electrophysiological study that combines i) atrial structure and fibrosis distribution from clinical MRI and ii) modeling of atrial electrophysiology, could be used to predict: (1) how fibrosis distribution determines the locations from which paced beats degrade into AF; (2) the dynamic behavior of persistent AF rotors; and (3) the optimal ablation targets in each patient. Four MRI-based patient-specific models of fibrotic left atria were generated, ranging in fibrosis amount. Virtual electrophysiological studies were performed in these models, and where AF was inducible, the dynamics of AF were used to determine the ablation locations that render AF non-inducible. In 2 of the 4 models patient-specific models AF was induced; in these models the distance between a given pacing location and the closest fibrotic region determined whether AF was inducible from that particular location, with only the mid-range distances resulting in arrhythmia. Phase singularities of persistent rotors were found to move within restricted regions of tissue, which were independent of the pacing location from which AF was induced. Electrophysiological sensitivity analysis demonstrated that these regions changed little with variations in electrophysiological parameters. Patient-specific distribution of fibrosis was thus found to be a critical component of AF initiation and maintenance. When the restricted regions encompassing the meander of the persistent phase singularities were modeled as ablation lesions, AF could no longer be induced. The study demonstrates that a patient-specific modeling approach to identify non-invasively AF ablation targets prior to the clinical procedure is feasible.

## Introduction

Atrial Fibrillation (AF), the most common arrhythmia, affects over 2 million people in the United States alone, and data suggests that its prevalence will continue to increase as the population ages[1,2]. Ectopic beats originating from the pulmonary veins (PVs) in the left atrium (LA) have been identified as a trigger that initiates AF[3]. Catheter-based ablation, the delivery of heat to destroy the ability of cardiac tissue to generate and conduct electrical signals locally, has emerged, over the last decade, as a promising treatment option; the procedure has successfully targeted the AF trigger via PV electrical isolation[4]. However, only a 70% success rate in achieving freedom from AF has been reported with this approach[5].

In an attempt to increase the success rate of the therapy, recent ablation strategies have begun to target atrial tissue in the LA wall as the substrate that perpetuates AF. These strategies include substrate ablation guided by the spatial distribution of complex fractionated atrial electrograms[6] and/or dominant frequencies[7]. A different ablation strategy has recently emerged[8], which involves identifying and ablating AF localized rotors. The variable success rates of these strategies have highlighted the fact that identifying which components of the atrial substrate sustain AF is critical for the correct identification of the optimal targets for ablation.

Clinical evidence has demonstrated that the extent of atrial fibrosis is correlated with both AF incidence[9] and recurrence after ablation[10], indicating its critical role in AF pathogenesis. Recent studies have also shown that the spatial distribution of fibrosis impacts AF dynamics[11,12], suggesting that the unique distribution of atrial fibrosis in each patient may govern the location of AF rotors, and could therefore potentially be used to identify critical targets for AF ablation[8], using a patient-specific approach.

In this study we aimed to provide a proof-of-concept that patient-specific virtual electrophysiological study that combines i) the anatomical structure and morphology of the patient atria and the unique distribution of atrial fibrosis as quantified from clinical MRI scans in vivo, and ii) computer modeling of electrophysiology of the atria could be used to predict: (1) how the unique patient-specific fibrosis distribution determines the locations from which pacing stimuli will degrade into reentrant activity in the fibrotic substrate; (2) the dynamic behavior of persistent AF rotors resulting from the presence of fibrosis in the individual atrial models; and (3) the optimal locations of ablation of the fibrotic substrate in each patient. Our study focuses exclusively on AF dynamics and ablation under the conditions of fibrotic remodeling, contributing both mechanistic insight and potential application in the clinical procedure of ablation.

## Methods

### Contrast-enhanced 3D MRI and Patient Atria Geometry Reconstruction

Researchers at the University of Utah have pioneered MRI-LGE methodology[13] to quantify the extent of atrial fibrotic remodeling. Our patient data were sourced from this clinical center. While recent clinical studies [14] report such MRI-LGE patient image acquisition at an average resolution of 1250×1250×1500μm^3^, such resolution is inadequate for constructing predictive patient-specific atrial models. Since the present research aimed to explore the application of the virtual electrophysiology approach in patients with different degrees of atrial fibrosis, navigating the constrains of MRI-LRE image acquisition for the goals of this study, four patient atrial scans at average resolution of 667×667×1500μm^3^ that could be used for atrial model reconstruction were provided to us for the study. Each of these four patients had an amount of left atrial (LA) wall enhancement (i.e. amount of atrial fibrosis) belonging to one of four groups[13]: Utah I (≤5%), Utah II (>5% to ≤20), Utah III (>20% to ≤35), or Utah IV (>35); the amount of LA fibrosis in the four chosen patients was quantified as 0.8%, 18.0%, 22.8%, and 42.0%. Patient ages and genders are included in Table 1.

**Table 1:** Patient data for the four atrial models.

	Patient’s Age	Patient’s Gender	% Fibrosis
Utah I	46.4	Female	0.8%
Utah II	60.7	Female	18.0%
Utah III	55.2	Female	22.8%
Utah IV	73.8	Female	42.0%

**Fig 1:**
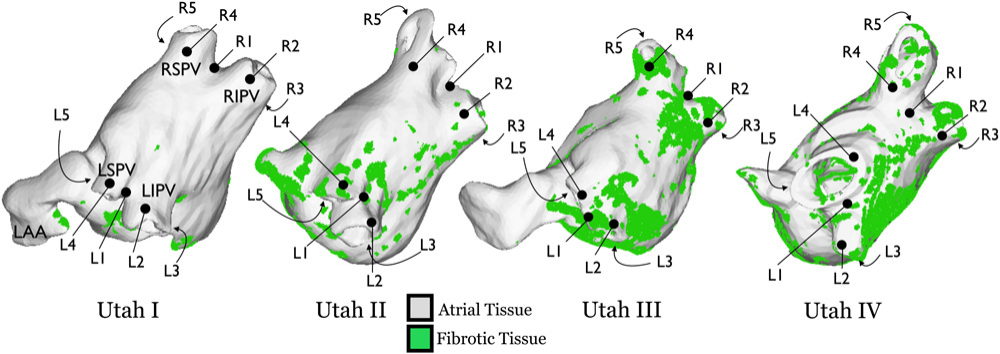
Patient-specific distributions of LA fibrosis for substrates Utah I—IV. Pacing locations in the left PVs (L1–L5) and right PVs (R1–R5) are indicated for each substrate. Anatomical locations for the left superior PV (LSPV), left inferior PV (LIPV), right superior PV (RSPV), right inferior PV (RIPV), and LA appendage (LAA) are indicated in the left-most substrate; all substrates are presented in identical orientations.

To reconstruct atrial anatomy, epicardial and endocardial atrial borders were manually contoured using Corview (MARREK Inc., Salt Lake City, UT, USA). The relative extent of LA wall enhancement was quantified with a threshold-based algorithm utilizing pixel intensities from atrial tissues[15]. LGE-MRI image segmentation and interpolation was used to produce a high-resolution image of the LA wall with accurate fibrotic lesion distribution for each of the four patients (Fig. 1); previous research has demonstrated that LGE-MRI enhancement correlates with regions of fibrosis identified by histological examination[16].

### Modeling of Atrial Electrophysiology

A finite element tetrahedral mesh was generated from each of the segmented image stacks[17]. Ensuring computational tractability in this study with an extensive number of simulations, we modeled only the LA; the latter choice was motivated by the fact that clinical evaluation of patients for AF ablation at the University of Utah center was based on MRI-LGE quantification of fibrosis in the LA only[10].

The patient-specific fiber orientation was estimated as previously described in detail[15,18]. Specifically, the estimation involved the use of a human atrial atlas data; using over 100 atrial geometrical landmarks, a 3D variant of the thin plate spline transformation was used in combination with large deformation diffeomorphic metric mapping to morph the atrial atlas geometry such that it closely matched the patient atria. Conductivities of 0.384 S/m in the primary myofiber direction and 0.0474 S/m in the cross-fiber direction were assigned such that an effective conduction velocity (CV) of 55 cm/s was achieved; detailed description of the rationale for this choice is presented in our previous publications[12,15].

Non-fibrotic regions of the tissue were represented with a model of the human atrial action potential under AF conditions[19] developed by Krummen et al[20] on the basis of AF patients electrophysiological data. Detailed description of the methodology for modeling fibrotic regions can be found in our previous publications[12,15]. This methodology is distinctly different from previous attempts to model fibrosis in patient-specific models, where fibrotic regions were represented by higher conduction anisotropy ratios [21,22]; the present modeling approach is a more comprehensive representation of the structural remodeling that takes place in the fibrotic myocardium. To briefly recap, gap junction remodeling due to connexin 43 protein down-regulation and lateralization was incorporated, with values of conductivities derived from experimental data. Diffuse and patchy collagen distributions were also included in the fibrotic lesions of the model. Diffuse collagen was modeled using 3D element decoupling to account for fine conduction barriers, where insulating sheets were randomly seeded and grown along element faces parallel to fiber orientation in the fibrotic regions. Patchy collagen was represented as passive insulators interrupting both transverse and longitudinal connections between cells. Finally, myocytes in the fibrotic regions had altered action potential duration (APD) resulting from myofibroblast proliferation[23–27] that leads to myocyte-fibroblast coupling, modeled as described previously in our single cell studies, including comparisons with experimental data [28,29]. In the atrial models, myofibroblast properties were assigned to 1% of the volume of the fibrotic regions, at random distribution, within the patient-specific fibrotic regions. Experimental evidence, including very recent work [30], supports the notion that myofibroblasts can alter the APD of neighboring myocytes [31,32]; the resulting APD dispersion at the tissue level increases the susceptibility of a substrate to arrhythmia [33]. We have previously demonstrated that modeling myofibroblast influence via heterocellular coupling or via direct changes in myocyte repolarization currents, as arising from paracrine factors [31], results in indistinguishable electrical consequences [12], thus our simulation methodology is general in terms of representing the influence of myofibroblasts in adjacent myocardium.

Note that in our atrial models the size, location, and morphology of the fibrotic lesions were patient specific, while the myofibroblasts and collagenous septa were randomly distributed within the fibrotic lesion borders since such patient-specific data cannot be currently obtained in the clinic.

### Simulation Protocol and Data Analysis

Monodomain simulations were executed using the package CARP[34–36]. To investigate how the distribution of fibrosis determines whether dynamic pacing will degrade into reentrant activity and initiate AF, initially ten pacing locations were chosen in each model. These initial stimulus locations (Fig. 1) were distributed around the PVs (where most ectopic beats originate[3]). For each pacing location, a dynamic pacing protocol was used to assess arrhythmia inducibility, as performed clinically[8]. The protocol consisted of pacing starting at 365ms cycle length followed by several beats at decreasing cycle lengths until 260ms was reached. Sustained AF was defined as fibrillatory activity lasting for 10s after the last stimulus. A number of additional pacing locations were used as necessitated by simulation results (see Results).

### Identifying Rotors and Representing Ablation

In models in which AF resulted from PV pacing, the AF rotors behavior was examined by determining the dynamic locations of the phase singularities (rotor organizing centers) throughout the 3D atrial models over a period of 10s, and the phase singularity movement (i.e. trajectory through the tissue, typically precession) was quantified for each rotor, using the approach we have previously published[37], based on methodology by Gray et al[38]. Based on the analysis, ablation lesions were implemented to so that the lesions fully covered the confined 3D regions of precession of the persistent rotors phase singularities. Rotors were considered persistent if they lasted for more than 5 rotations. Lesions were modeled as one or more transmural regions[39] of inexcitable tissue; they were cylindrical to account for catheter tip shape, and 7mm in diameter (within the range of clinical ablation lesions[8]). Arrhythmia inducibility was tested from numerous locations, both in the PVs and outside, after the implementation of each ablation lesion.

All simulations were blinded to patient history.

## Results

### Arrhythmia Inducibility in Patient-Specific LA Models

**Fig 2:**
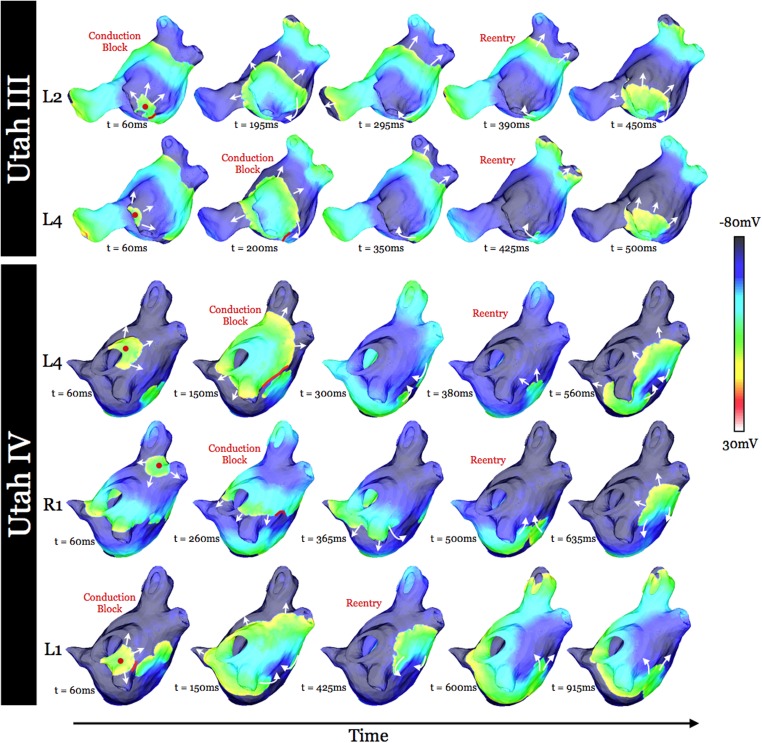
Transmembrane potential maps at five time instants, documenting AF initiation in substrates Utah III (top two rows) and IV (bottom three rows) from different PV pacing locations (as noted at left). Lines of block are marked in red. White arrows indicate direction of propagation. Red dots (left-most column) indicate pacing location.

Our virtual electrophysiology study demonstrated that neither the Utah I nor the Utah II models resulted in arrhythmia following pacing from any of the initial ten PV locations. The Utah III substrate developed a sustained AF following pacing from two of the initial ten PV locations; those were pacing sites L2 and L4 (as marked in Fig. 1). Pacing from three of the ten PV locations (L1, L4, and R1, Fig. 1) resulted in sustained AF in Utah IV. Fig. 2 presents the events leading to the formation of the first cycle of sustained reentry resulting from dynamic pacing at locations that initiated AF in models Utah III and IV. In each case, unidirectional conduction block (red lines in Fig. 2) occurred resulting from either the stimulus-induced wavefront encountering refractory tissue (Utah IV: R1) or the collision of the stimulus-induced wavefront with the wavefront elicited by the preceding stimulus (Utah III: L2 and L4; Utah IV: L1 and L4). The latter case was possible because of the slow and discontinuous conduction that occurred in the large fibrotic lesions in substrates Utah III and IV, resulting in the wave taking a propagation direction different from that in non-fibrotic tissue. Following unidirectional conduction block in substrates Utah III and IV, a reentrant circuit was formed, leading to the onset of sustained AF.

In all cases, simulation outcomes matched patient arrhythmia outcome. As it could be expected based on the amount of fibrosis in the patient atria, patients from which the Utah I and II LA models were generated did not suffer from persistent AF, while the patients whose scans were used to create the Utah III and IV LA models both did.

### Distribution of Atrial Fibrosis Determines the Locations from which Paced Beats Degrade into Persistent AF

Clinical findings indicate that not all atrial ectopic beats result in initiation of reentrant arrhythmia, even in patients in whom AF was previously documented[3]. This is consistent with the results of our virtual electrophysiological study, where pacing from only a few of the 10 initial PV locations resulted in AF in substrates Utah III and IV. We aimed to understand why ectopy from some sites triggers arrhythmia, while from others it does not.

**Fig 3:**
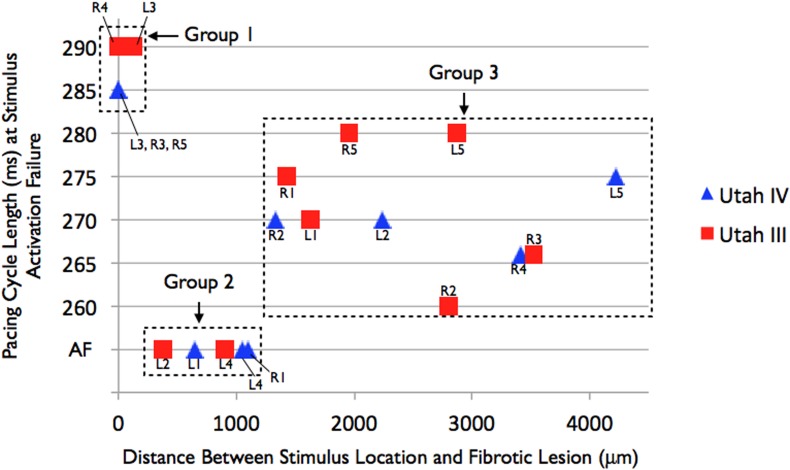
Plot of the distance between each pacing site and its closest fibrotic lesion vs. the pacing cycle length at which a stimulus either failed to excite the tissue or resulted in AF in substrates Utah III (red squares) and Utah IV (blue triangles). Stimulus locations are labeled for each data point. Groups 1–3 are indicated for discussion purposes. Distances between pacing site and fibrotic lesions cannot be inferred from Figs. 1 or 2 due to the limitations in presenting 3D information in a 2D figure.

The results from our simulations revealed that at all locations from which AF was initiated in substrates Utah III and IV, AF initiation occurred following the entire pacing protocol (starting at cycle length of 365ms and proceeding down to 260ms). At locations from which AF was not induced, only part of the pacing protocol was executed, since a stimulus from the pacing train failed to elicit propagation before the pacing protocol was completed (that is, prior to reaching 260ms cycle length). Given that repolarization dynamics throughout the fibrotic LA are non-uniform, we sought to determine how a PV pacing location’s relation to the region of fibrosis affects the ability of a stimulus from that location to elicit excitation in the Utah III and IV models. The results were analyzed by plotting, in Fig. 3, the distance between each PV pacing site and its closest fibrotic lesion against the pacing cycle length at which excitation failed at that pacing site. At PV locations where AF was initiated, the cycle length was 260ms, the shortest cycle length in the pacing protocol, as already mentioned above. Fig. 3 demonstrates a clustering of three groups of data points: Group 1, PV pacing locations for which there was a fibrotic lesion in close proximity (<378μm) and for which stimuli with cycle lengths of 285–290ms in the pacing protocol failed to excite the tissue; Group 2, PV pacing locations that were between 378 and 1052μm from fibrotic lesions and for which sustained AF resulted following the execution of the entire pacing protocol; and Group 3, stimulus locations that were far from fibrotic lesions (>1052μm) and for which stimuli with cycle lengths of 260–280ms failed to excite the tissue. For each of these groups, we investigated the underlying mechanisms that created these distinctions in behavior:

Group 1. In Group 1 simulations, the PV pacing locations were either located within fibrotic lesions or in an immediate proximity to fibrotic lesions. Partial sodium channel inactivation precipitated by the presence of myofibroblasts (seeded at a density of 1%) resulted in some post-repolarization refractoriness[12] in the fibrotic regions. Therefore, short pacing cycle lengths in our pacing protocol (shorter than 285ms) were unable to full-blown propagating activations (thus AF) from PV pacing locations within or in close proximity to the fibrotic lesions.

**Fig 4:**
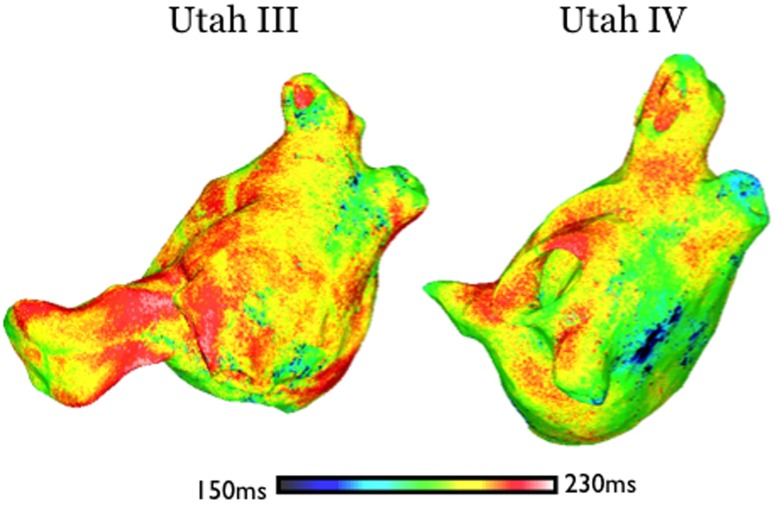
Transmembrane potential maps at four time instants in substrates Utah III (top row) and Utah IV (bottom row) following pacing from a “prime” region outside the PVs (pacing sites marked by red arrows).

In Group 2, PV pacing locations were between 378 and 1052μm from fibrotic lesions (intermediate distances, which we term“prime tissue”); sustained AF resulted following the execution of the full pacing protocol from these pacing locations. At these locations, there is no partial sodium channel inactivation (pacing locations are sufficiently far for that from the fibrotic regions). However, these regions are close enough to the fibrotic regions to be characterized with APD shortening stemming from electrotonic interactions with the fibrotic regions. The spatial dispersion of APD that occurs throughout in Utah III and IV substrates as a result of remodeling and fibroblast proliferation in the fibrotic lesions is shown in Fig. 4; these maps indicate significant APD shortening and dispersion within the fibrotic regions that descreased with the distance away from the fibrotic regions. The average APD at Group 2 PV pacing locations was found to be 9.5ms shorter than the average APD of Group 3 PV pacing locations since tissue that is closer to fibrotic lesions experienced a greater degree of APD shortening. Thus, pacing stimuli at short cycle lengths from PV locations that fell within this mid-range of distances to fibrotic lesions (378–1052μm) encountered tissue that was excitable, allowing for propagation through it, so that the paced-induced activation could then encounter fully the dispersion of APD within the fibrotic regions and degenerate into AF.

In Group 3 simulations, PV pacing locations were far from fibrotic lesions (> 1330μm) and did not experience the same extent of APD shortening as those that were closer, as evidenced by the average APD values presented above. Therefore, stimuli at short pacing cycle lengths encountered tissue in refractory state and could not elicit activation.

Based on the finding that stimuli that were located at a distance between 378 and 1052μm from fibrotic lesions could initiate AF in models Utah III and IV, the distance between each point in the non-fibrotic tissue and the closest fibrotic lesion was calculated to determine what locations would fall within the range of these “sweet spot” distances to fibrotic lesions and could therefore be considered “prime spots” for AF initiation. We found that “prime” locations comprise 3.56% and 4.43% of LA volume (including the PVs) in the Utah III and IV models, respectively. A disproportionately large amount of this “prime” tissue was localized in the PVs, 19.78% and 32.56% in the Utah III and IV substrates, respectively, although the PVs comprised only 14.9% and 22.93% of total tissue volume, respectively, in these models. It is important to note that it is unclear whether this finding will hold true in other patient atria with persistent AF and fibrosis, or whether it is specific to the patient atria used here because of the particular distribution of fibrosis in proximity of the PVs.

**Fig 5:**
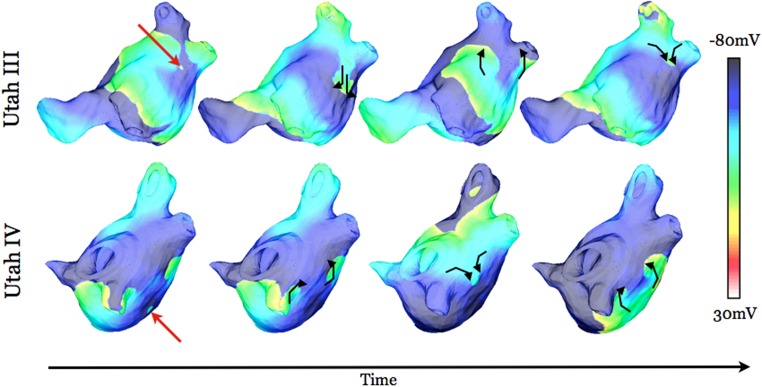
Maps of APD distribution for atrial models Utah III (left) and Utah IV (right).

Building on this finding, we conducted 10 additional simulations for each of the two atrial models, where pacing trains were delivered from 10 locations in “prime” tissue *outside of the PV regions* in substrates Utah III and IV to test AF inducibility and the presence of “sweet spot for AF induction”; consistent with our predictions above, sustained AF was initiated by pacing from all of these locations. Data from representative simulations are shown in Fig. 5.

### Distribution of Atrial Fibrosis Determines Persistent AF Rotor Location and Meander

**Fig 6:**
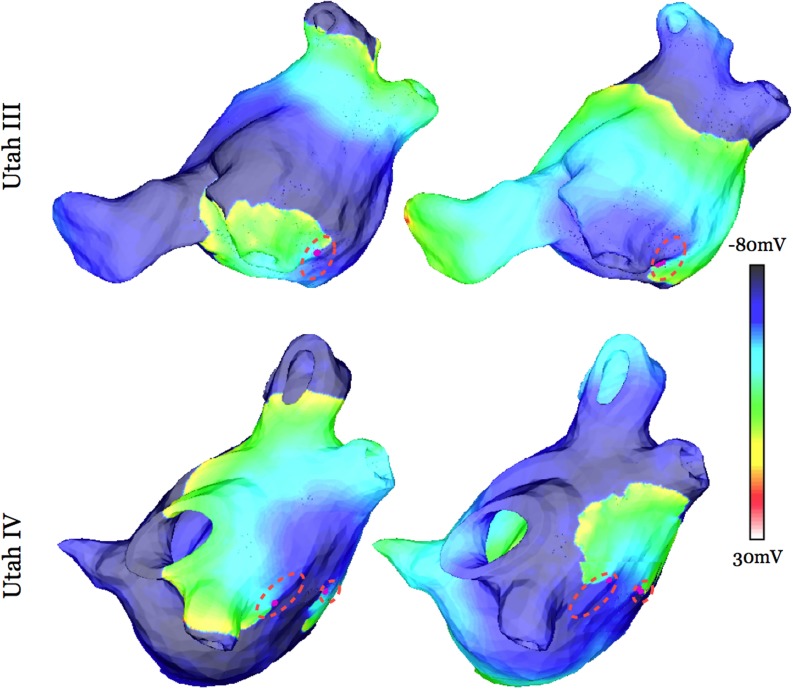
Epicardial transmembrane potential maps at two time instants in atrial models Utah III (top row) and Utah IV (bottom row) demonstrating an instant location (pink dot) of the phase singularity at the time instant of the transmembrane potential map, as well as the outline of the confined region of precession (red dashed line) of the persistent phase singularities. The persistent phase singularities meandered only within (not around) the regions outlined with red dashed lines.

Phase singularities were calculated throughout the volume of the atrial models in all of the cases in which pacing resulted in persistent AF initiation. In both Utah III and Utah IV models AF, was characterized with the formation of persistent rotors, which lasted for the duration of the AF simulations, as well as other “breakoff” fibrillatory activity, the phase singularities of which appeared and vanished fairly quickly. Importantly, once AF was initiated, the persistent rotors moved (in a precession-like way) within the same confined regions in each model, regardless of the pacing location from which AF was induced, indicating that the patient-specific distribution of fibrosis, rather than the location of atrial trigger, was the most important factor governing AF persistent rotor location(s). In the Utah III substrate (Fig. 6, top row), as shown in the epicardial surface map, one persistent rotor was found to move around within an approximately oval region of tissue with long and short diameters of 13.2mm and 6.7mm, respectively (calculated persistent phase singularities locations at the given instant of time are presented in Fig. 6 by the pink dots on the epicardial surface; confined regions of precessions are outlined by red dashed line). In the Utah IV substrate, there were two persistent rotors, one moving cyclically within a larger oval region of tissue with long and short diameters of 13.7mm and 6.2mm, respectively, and another, the movement of which took place in a smaller, approximately circular, region of tissue that was about 6.6mm in diameter (Fig. 6, bottom row). Note that in the Utah III and IV models, where AF is maintained due to fibrotic remodeling only, we did not find appreciable differences in the way the phase singularities associated with the same rotor moved on the endocardial and epicardial surfaces. This is consistent with findings by Arevalo et al [40] regarding reentrant wave filament behavior associated with infarct-related ventricular tachycardia, and the anchoring of reentrant waves found in 2D models of atrial fibrosis [24].

**Fig 7:**
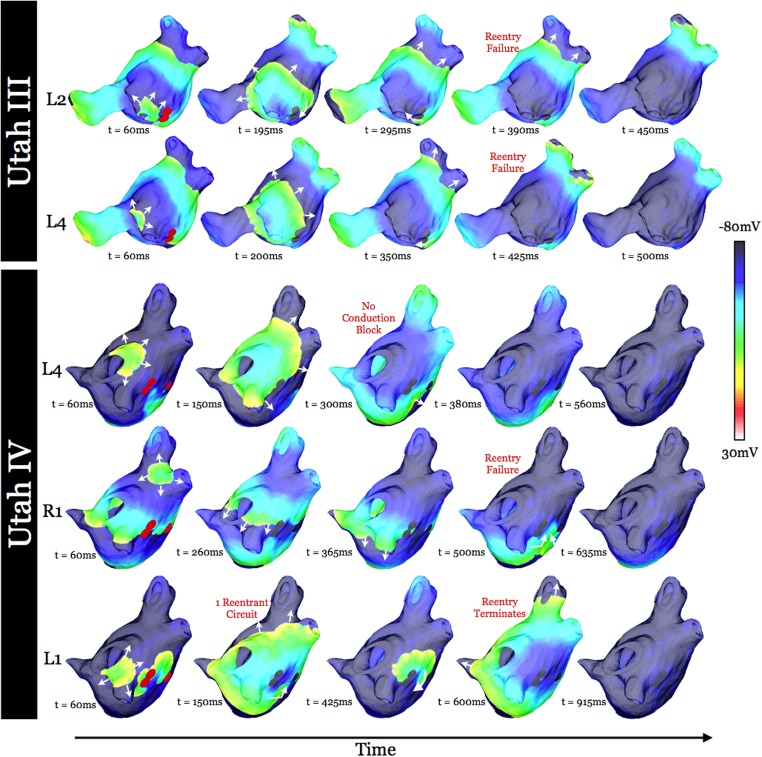
Transmembrane potential maps at five time instants in substrates Utah III (top two rows) and IV (bottom three rows) from different pacing locations (as noted at left) with ablation lesions modeled. Filled red circles (left-most column) represent the extent of the ablation lesions. White arrows indicate direction of propagation. Time instances and pacing locations are identical to those in Fig. 2.

Ablation was applied, as described in Methods, to the confined regions within which the persistent phase singularities moved. Snapshots of transmembrane potential distributions at time instants throughout simulations with ablation lesions modeled are shown in Fig. 7; the selected time instants are identical to those in Fig. 2 to allow for comparison. The implementation of ablation consisting of two overlapping circular lesions (indicated in red in the left-most images in Fig. 7), fully covering the long diameter (13.2mm) of the region of mother rotor phase singularity movement was necessary to result in AF non-inducibility in the Utah III substrate following pacing from both the L2 and L4 locations. In the Utah IV substrate, two separate ablations were implemented to render inexcitable the regions of movement of the two persistent phase singularities. The smaller region was targeted with one circular ablation, while the larger region was targeted with two overlapping circular lesions to again fully cover the long diameter of the region confining the persistent phase singularity movement (13.7mm). The ablations (indicated in red in the left-most images in Fig. 7) resulted in AF non-inducibility. It was not possible to achieve AF non-inducibility with fewer ablation lesions.

Finally, we performed limited parameter sensitivity analysis (as to keep the simulation study tractable) to determine how a change in electrophysiological parameters affected the dynamics of phase singularities in the Utah III model. To examine the effect of conduction velocity on the locations of persistent phase singularity, longitudinal and transverse conductivity values in the atrial model were increased by 50%, and the simulations with this model repeated. As a result, the mean location of the persistent phase singularity changed by 0.5149 cm (the persistent rotor average cycle length decreased by 14 ms). Additionally, we changed APD duration in the fibrotic regions by increasing the density of the myofibroblasts by 20%, and repeating the simulations with several random distributions. This resulted in a mean phase singularity location change of 0.44 cm (AF persistent rotor cycle length decreased by 15 ms). These simulations demonstrated that the patient-specific distribution of fibrosis was the primary determinant of the locations of the persistent AF rotors.

Note that in this study it was impossible to compare the predicted optimal ablation lesions with clinical ones, the reason being that patients with significant fibrosis such as those in categories Utah III and IV are counter-indicated for atrial ablation because 1) PV isolation, the standard approach, is insufficient to terminate AF and 2) it is currently unknown where to ablate in the fibrotic substrate in these patients. Indeed, it is our hope that further developments of the patient-specific approach for predicting the optimal ablation targets presented here will be translated to the clinic so that patients with fibrotic atria and persistent AF could be successfully ablated and rendered free of AF based on simulation predictions of rotor dynamics in the specific fibrotic substrate.

## Discussion

### AF phase singularity dynamics and the optimal targets of AF ablation

In this study we developed the first time MRI-based patient-specific models of AF from patients with different burden of atrial fibrosis. The patient-specific aspects of the models were atrial geometry and the specific fibrosis distribution, as reconstructed from clinical LGE-MRI scans. Four models were developed for this study, each with fibrosis burden falling in one of the four Utah fibrosis categories. In two of the models, with fibrosis burden categories Utah III and Utah IV, AF was induced from numerous pacing locations in the simulations. The dynamics of the persistent phase singularities during AF induced from those pacing locations was evaluated. In these two AF models, simulations demonstrated that the unique distribution of fibrosis in the patient atria plays an important role in the dynamics of the patient’s AF persistent phase singularities. In all cases, the persistent phase singularities moved within confined regions in the atria, in a precession-like movement. This localization was independent of the pacing locations from which AF was induced, whether PVs or other locations in the atria outside the fibrotic regions.

When “ablation” was performed in the patient-specific atrial models by rendering each of the confined regions of persistent phase singularities movement non-conductive (i.e, making them ablation lesions), AF became non-inducible from any of the tested pacing locations in the atria. These results agree with clinical findings by Narayan and co-workers[8], where the region of precession of the AF rotor’s organizing center was a successful ablation target. Also consistent with our finding that the implementation of 1 to 2 ablation lesions led to AF non-inducibility, Narayan et al. reported that an average of 2.1 ± 1.0 electrical rotors were observed in patients with sustained AF.

The findings could have an important implication: since the patient-specific fibrosis distribution was found here to be the main determinant of the spatial localization of the organizing centers of persistent reentries in AF, then it is possible to envision that patient-specific models of the fibrotic atria could be constructed to predict, non-invasively, the optimal AF ablation targets using models reconstructed from clinical MRI-LGE scans.

To further demonstrate the important role of the specific fibrotic distribution in the localization of the persistent phase singularities in the patient-specific atrial model, a limited (as to ensure computational tractability) electrophysiological parameter sensitivity analysis was conducted. While changes in electrophysiological parameters would be expected to alter the dynamics of AF, these changes translated into only a small change in the localization of the AF persistent phase singularities. This change in the localization could be considered insignificant if it would be at a distance covered by a clinical ablation lesion [41]. For instance, the simulations demonstrated that a change in CV by 50% resulted in close to 0.5cm change in the mean location of the persistent phase singularity in Utah III model, well within the limits of a clinical lesion. Thus, patient-specific variability in electrophysiological parameters might play a much smaller role in determining the spatial localization of the organizing centers of persistent reentries in AF, and thus the optimal targets of persistent AF ablation in the fibrotic atria. These findings are in agreement with results by Arevalo et al [40], where the simulated (in an image-based ventricular model) infarct-related ventricular tachycardia organizing centers of reentry were found to always be localized in the infarct gray (or border) zone, with the specific location determined predominantly by the morphology of the border zone, as shown by the electrophysiological parameter sensitivity analysis.

Should the present findings be repeated in a number of patient-specific models, they could have important implications for the clinical translation of patient-specific atrial modeling. The virtual electrophysiological study using patient-specific atrial models could provide a novel way to identify regions of meander of persistent phase singularities based on the individual spatial distribution of fibrosis. This study therefore presents the proof-of-concept of a non-invasive approach to the identification of the optimal ablation targets for persistent AF in the fibrotic atria. In its translation to the clinic, we envision that the approach will entail the use, prior to the clinical procedure, of an MRI-based subject-specific multiscale electrophysiological model of the fibrotic atria to analyze AF dynamics and rotor meander, and to determine the optimal targets of ablation. Once the optimal targets of ablation are determined and visualized by the present approach, we envision that ablation delivery for AF termination could be swift and precise, eradicating, with a minimal number of lesions, all rotors in the fibrotic substrate. This could dramatically improve the efficacy of ablation, increase the tolerance for the procedure, and reduce post-procedure complications and long-term deleterious effects resulting from the lengthy invasive mapping and the numerous unnecessary ablation lesions.

### Pacing locations and inducibility of AF

The present study also examined how the patient-specific fibrosis distribution determines whether pacing stimuli result in persistent AF, and from which specific atrial locations. The simulations demonstrated that: (1) The mechanisms, which determine whether pacing from a given atrial site will degrade into AF, operate in a distance-to-fibrosis-dependent fashion, with pacing from locations only in the mid-range of distances (378–1052μm) to fibrotic lesions resulting in sustained AF, and 2) A disproportionate amount of all non-fibrotic tissue that falls in the mid-range (i.e arrhythmogenic “sweet spot”) distance to fibrotic lesions is located in the PVs.

In this study, two out of ten PV stimulus locations resulted in AF initiation in the Utah III substrate (with 22.8% fibrosis), while three out of ten PV stimulus locations caused AF in the Utah IV substrate (with 42.0% fibrosis); no PV stimuli caused AF in the Utah I and II models. This finding demonstrates a correlation between the amount of fibrosis and the probability of a trigger initiating AF; it is consistent with the correlation found between AF incidence and percent fibrosis in the LA[9], the underlying cause of which has remained unknown. Our study findings suggest that AF incidence may be higher in patients with more fibrosis due to the fact that larger degrees of fibrosis cause locations in the substrate to become “prime” for triggering AF. Indeed, we demonstrated that “prime” trigger locations comprise 3.56% and 4.43% of the Utah III and IV substrates by volume, respectively, of which disproportionate amounts were located in the PVs. The concept of “prime” tissue may help explain the clinical finding that triggered activity, which can remain frequent years after linear LA ablation, often does not initiate AF[42]. Should ablation create an electrical barrier that prevents propagation from “prime tissue”, substrates could be rendered non-inducible to AF, despite the presence of triggered activity.

Traditionally, AF treatments have either been aimed at suppressing atrial triggers that initiate AF[4], or at modifying the substrate that sustains it[6,7]. In this study, however, the spatial distribution of atrial fibrosis was found both to determine whether ectopy initiates AF and to govern the dynamics of the resulting AF rotors. The fact that the distribution of atrial fibrosis modulates both AF initiation and its maintenance creates a new paradigm for AF treatment, in which one treatment strategy could possibly target both AF-triggering and-perpetuating mechanisms.

**Study limitations**. Because fibrosis was identified in the clinical images used here in LA only, and because this study focused only on the role of fibrosis on AF reentrant activity, for computational tractability simulations used only the LA to determine AF rotor dynamics and perform ablation. The methodology would not change when modeling both atria; similarly, the insights obtained here will remain valid if the RA is non-fibrotic. Should presence of fibrosis be demonstrated clinically by LGE-MRI imaging in the RA as well, persistent rotors might also occur in the RA, which would be consistent with findings by Narayan et al [43] that over 30% of all rotors sustaining persistent AF are found in the RA; this will necessitate ablation lesions there as well. Another limitation of the study is the small number of patient scans due to the low resolution of the scans. However, image resolution is likely to improve with time and advances in MRI technology, which will improve the utility of our approach. Likewise, the fact that an ionic model of chronic AF was used for all patient models is a limitation. Finally, consistent with the goals of this study, our model does not include intrinsic APD gradients that might exist in the patient LA non-fibrotic regions; the latter have been the subject of numerous experimental and simulation studies (see reviews, some very recent [44–46] as well as new studies [47]). It is possible that different sets of mechanisms dominate AF dynamics under different conditions. Thus, the concept of “prime tissue” in our study may also be limited; while it may control the minimum pacing cycle rate that can be obtained at a certain location, reentrant activity may also depend on heterogeneous tissue properties elsewhere.
