# Correction: Virtual Electrophysiological Study of Atrial Fibrillation in Fibrotic Remodeling

**DOI:** 10.1371/journal.pone.0156189

**Published:** 2016-05-19

**Authors:** Kathleen S. McDowell, Sohail Zahid, Fijoy Vadakkumpadan, Joshua Blauer, Rob S. MacLeod, Natalia A. Trayanova

The caption descriptions for Figs 4 and 5 are switched. The authors have provided a corrected version here.

**Fig 4:**
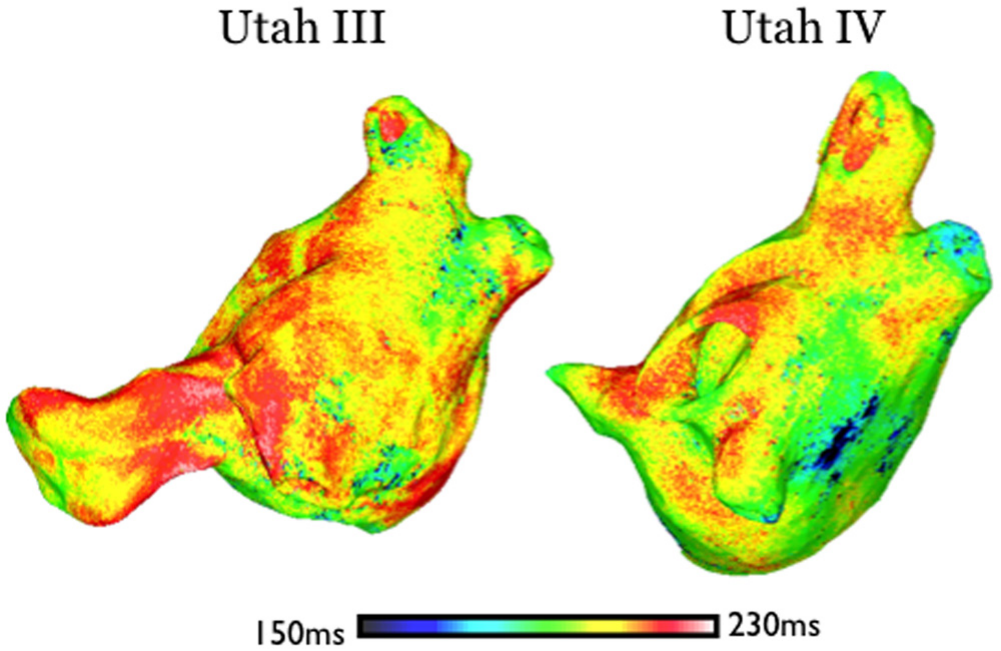
Maps of APD distribution for atrial models Utah III (left) and Utah IV (right).

**Fig 5:**
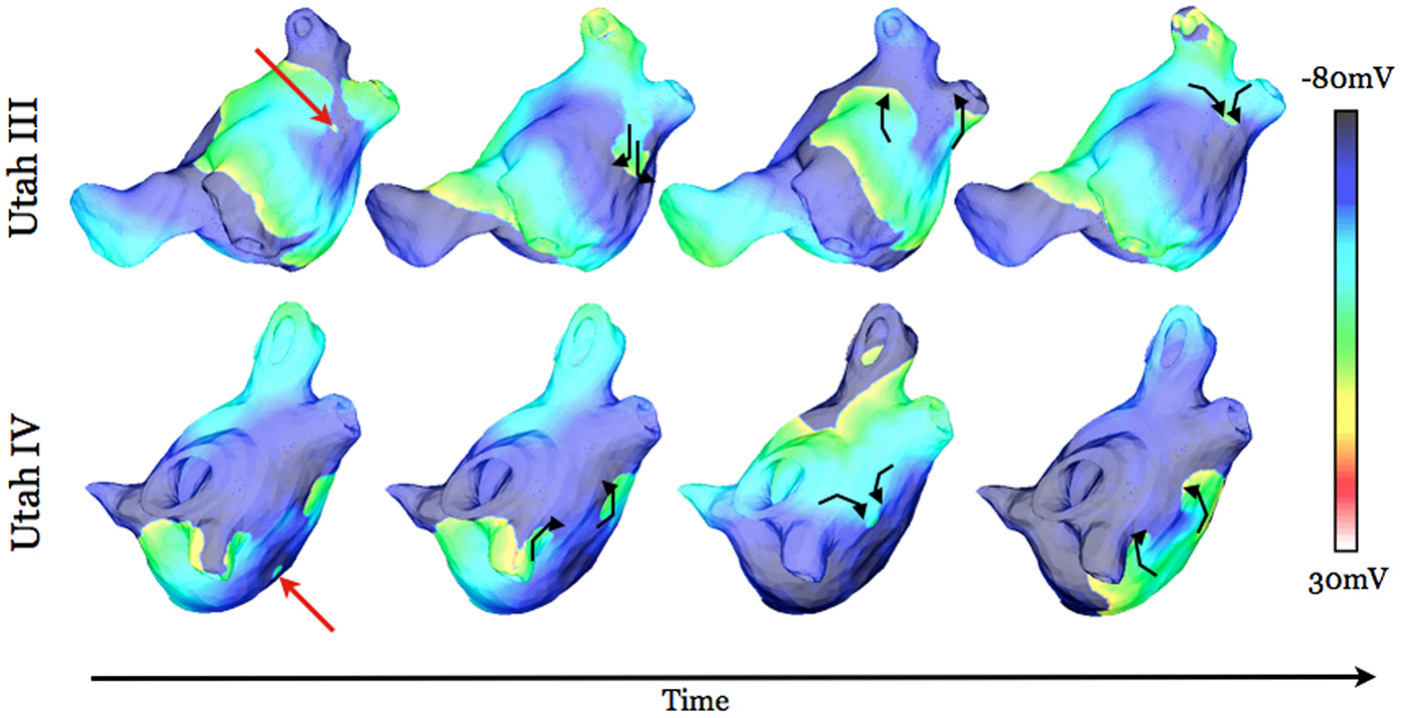
Transmembrane potential maps at four time instants in substrates Utah III (top row) and Utah IV (bottom row) following pacing from a “prime” region outside the PVs (pacing sites marked by red arrows).
